# Correction: Xiao, H., et al. Social Distancing among Medical Students during the 2019 Coronavirus Disease Pandemic in China: Disease Awareness, Anxiety Disorder, Depression, and Behavioral Activities. *Int. J. Environ. Res. Public Health* 2020, *17*, 5047

**DOI:** 10.3390/ijerph18010148

**Published:** 2020-12-28

**Authors:** Huidi Xiao, Wen Shu, Menglong Li, Ziang Li, Fangbiao Tao, Xiaoyan Wu, Yizhen Yu, Heng Meng, Sten H. Vermund, Yifei Hu

**Affiliations:** 1 Department of Child and Adolescent Health and Maternal Care, School of Public Health, Capital Medical University, Beijing 100069, China; xhd19988023@163.com (H.X.); nmg_sw026@163.com (W.S.); limenglong_ph@163.com (M.L.); 15622764530@163.com (Z.L.); 2 Department of Maternal, Child and Adolescent Health, School of Public Health, Anhui Medical University, Hefei 230032, China; fbtao@ahmu.edu.cn (F.T.); xywu85@126.com (X.W.); 3 Department of Child and Women Health Care, School of Public Health, Tongji Medical College, Huazhong University of Science and Technology, Wuhan 430030, China; yuyizhen650@163.com (Y.Y.); menghengmay@hust.edu.cn (H.M.); 4 Department of Epidemiology of Microbial Diseases, Yale School of Public Health, New Haven, CT 06520, USA; sten.vermund@yale.edu; 5 Department of Pediatrics, Yale School of Medicine, New Haven, CT 06520, USA

In our recently published article [1], there were mistakes in Table 1 as published. The corrected Table 1 appears as below, which includes a typographical error noted by Dr Batra. Moreover, to avoid confusion, we keep the percentage data in the bracket only in the “correct answer” column. Our footnote defines “correct rate” as: correct answer rate = sum of correct answers/total number of answers.

After the corrections, we calculated new *p* values for the correct answer rate (also relevant to Table 1). A correction has been made to *3.1* Preventive Knowledge, Anxiety Disorders, and Depression; one of the *p* values that was *p* = 0.037 is revised as *p* < 0.001 (our update of Table 1). The other correction has been made to *3.4.* Predictors of Anxiety Disorder and Depression Using Multivariable Logistic Regression and Path Analysis; 426 (48.7%) is revised as 426 (45.7%), also a typographical error. We are sorry for the mistakes and appreciate Dr. Batra bringing them to our attention. Our scientific conclusions are unaffected.

## Figures and Tables

**Table 1:** Coronavirus disease (COVID-19) preventive knowledge, and depression, anxiety disorders of college students categorized by socio-demographic characteristics (*N* = 933).

Characteristics	n (%)	Knowledge			Anxiety Disorder		Depression	
		Score (0–4)	Correct Answers Rate (%)	*p*	Prevalence (%)	*p*	Prevalence (%)	*p*
Sex				0.005		0.015		<0.001
Male	279 (29.9)	3.12	78.0		35 (12.5)		47 (16.8)	
Female	654 (70.1)	3.28	82.0		125 (19.1)		189 (28.9)	
Age in years				0.011		0.58		0.335
17–24	755 (80.9)	3.2	80.0		132 (17.5)		196 (26.0)	
25 or older	178 (19.1)	3.37	84.3		28 (15.7)		40 (22.5)	
Grade of medical students				0.0001		0.30		0.57
Undergraduates	620 (66.5)	3.15	78.8		98 (15.8)		154 (24.8)	
MPH graduate students	228 (24.4)	3.4	85.0		46 (20.2)		63 (27.6)	
DrPh graduate students	85 (9.1)	3.34	83.5		16 (18.8)		19 (22.4)	
University				0.327		0.001		0.12
CCMU	558 (59.8)	3.21	80.3		77 (13.8)		131 (23.5)	
HUST	375 (40.2)	3.26	81.5		83 (22.1)		105 (28.0)	
Living residence				0.043		0.073		0.25
Village	350 (37.5)	3.16	79.0		70 (20.0)		96 (27.4)	
City	583 (62.5)	3.27	81.8		90 (15.4)		140 (24.0)	
Overall		3.23	80.8		160 (17.1)		236 (25.3)	

CCMC = Capital Medical University; HUST = Huazhong University of Science and Technology. MPH = Masters of Public Health degree; DrPh = Doctor of Philosophy degree. Correct answer rate = sum of correct answers/total number of answers.
