# Supplementary material for: Social Distancing among Medical Students during the 2019 Coronavirus Disease Pandemic in China: Disease Awareness, Anxiety Disorder, Depression, and Behavioral Activities
Source: Int J Environ Res Public Health. 2020 Jul 14;17(14):5047. doi: 10.3390/ijerph17145047 (PMC7399842; doi:10.3390/ijerph17145047)
Supplement: Supplementary file 1 [file ijerph-17-05047-s001.pdf]

Table S1. Preventive knowledge of COVID-19, comparing correct answer frequencies between the two universities ($N = 933$).

Questions	Answers	Number (%)			χ^2	p
		**Overall**	Beijing (558)	Wuhan (375)		
**How long is the incubation period of COVID-19?**	**1~14 days***	**907 (97.2)**	540 (96.8)	367 (97.9)	0.99	0.32
	15~21 days	24 (2.6)	16 (2.9)	8 (2.1)		
	22~30 days	2 (0.2)	2 (0.4)	0		
Which is not true about the mortality of COVID-19?	Wuhan is higher than other regions	153 (16.4)	93 (16.7)	60 (16.0)		
	Lower than flu*	626 (67.1)	360 (64.5)	266 (70.9)	4.18	0.04
	Lower than H7N9	51 (5.5)	31 (5.6)	20 (5.3)		
	Lower than SARS	103 (11.0)	74 (13.3)	29 (7.7)		
Which are susceptible groups for COVID-19?	Children	4 (0.4)	2 (0.4)	2 (0.5)		
	Young people	3(0.3)	3 (0.5)	0		
	Middle aged and elderly people	279 (29.9)	163 (29.2)	116 (30.9)		
	All people*	647 (69.3)	390 (69.9)	257 (68.5)	0.20	0.66
What drugs cure COVID-19 effectively?	Oseltamivir	87 (9.3)	49 (8.8)	38 (10.1)		
	Antibiotic	10 (1.1)	7 (1.3)	3 (0.8)		
	Glucocorticoid	2 (0.2)	1 (0.2)	1 (0.3)		
	No effective drugs*	834 (89.4)	501 (89.8)	333 (88.8)	0.23	0.63

Note: * means correct answer. The Chi-squared analysis is performed only between correct rates.

Table S2. Direct and indirect coefficient values in standardized estimation from the path analysis model ($N = 933$).

	**Factors**	**Direct coefficient**	**Indirect coefficient**	**Total coefficient**
Anxiety disorder	Negative thoughts or actions	0.87	-	0.87
Depression	Age (ref = age ≤ 24)	–0.07	0.06	–0.02
	Grade (ref = undergraduates)	0.07	–0.05	0.02
	Anger and quarreling behaviors	-	0.3	0.3
	Concerns about the COVID-19 epidemic	-	0.26	0.26
	Negative thoughts or actions	0.86	-	0.86
	Healthy lifestyle	–0.08	-	–0.08
	Anger and quarreling behaviors	-	0.29	0.29
	Concerns about the COVID-19 epidemic	-	0.26	0.26
	Longer video screen time	-	0.02	0.02
